# Mouse characteristics that affect establishing xenografts from hepatocellular carcinoma patient biopsies in the United States

**DOI:** 10.1002/cam4.4375

**Published:** 2021-12-24

**Authors:** Chenhui Zou, Imane El Dika, Koen O. A. Vercauteren, Marinela Capanu, Joanne Chou, Jinru Shia, Jill Pilet, Corrine Quirk, Gadi Lalazar, Linda Andrus, Mohammad Kabbani, Amin Yaqubie, Danny Khalil, Taha Mergoub, Luis Chiriboga, Charles M. Rice, Ghassan K. Abou‐Alfa, Ype P. de Jong

**Affiliations:** ^1^ Division of Gastroenterology and Hepatology Weill Medical College at Cornell University New York New York USA; ^2^ Laboratory of Virology and Infectious Disease The Rockefeller University New York New York USA; ^3^ Department of Medicine Memorial Sloan Kettering Cancer Center New York New York USA; ^4^ Department of Medicine Weill Medical College at Cornell University New York New York USA; ^5^ Department of Epidemiology and Biostatistics Memorial Sloan Kettering Cancer Center New York New York USA; ^6^ Department of Pathology Memorial Sloan Kettering Cancer Center New York New York USA; ^7^ Laboratory of Cellular Biophysics The Rockefeller University New York New York USA; ^8^ Department of Gastroenterology, Hepatology and Endocrinology Hannover Medical School Hannover Germany; ^9^ Memorial Sloan Kettering Cancer Center Sloan Kettering Institute New York New York USA; ^10^ Department of Pathology Center for Biospecimen Research and Development NYU Langone Health New York New York USA; ^11^ Present address: Institute of Tropical Medicine Antwerp Belgium; ^12^ Present address: Inserm UMRS1138 Functional Genomics of Solid Tumors (FunGeST) Paris France

**Keywords:** *Fah^−/−^* mice, human alpha1‐antitrypsin, nitisinone, PDX

## Abstract

**Background:**

Hepatocellular carcinoma (HCC) patient‐derived xenograft (PDX) models hold potential to advance knowledge in HCC biology to help improve systemic therapies. Beside hepatitis B virus‐associated tumors, HCC is poorly established in PDX.

**Methods:**

PDX formation from fresh HCC biopsies were obtained and implanted intrahepatically or in subrenal capsule (SRC). Mouse liver injury was induced in immunodeficient *Fah* ^−/−^ mice through cycling off nitisinone after HCC biopsy implantation, versus continuous nitisinone as non‐liver injury controls. Mice with macroscopically detectable PDX showed rising human alpha1‐antitrypsin (hAAT) serum levels, and conversely, no PDX was observed in mice with undetectable hAAT.

**Results:**

Using rising hAAT as a marker for PDX formation, 20 PDX were established out of 45 HCC biopsy specimens (44%) reflecting the four major HCC etiologies most commonly identified at Memorial SloanKettering similar to many other institutions in the United States. PDX was established only in severely immunodeficient mice lacking lymphocytes and NK cells. Implantation under the renal capsule improved PDX formation two‐fold compared to intrahepatic implantation. Two out of 18 biopsies required murine liver injury to establish PDX, one associated with hepatitis C virus and one with alcoholic liver disease. PDX tumors were histologically comparable to biopsy specimens and 75% of PDX lines could be passaged.

**Conclusions:**

Using cycling off nitisinone‐induced liver injury, HCC biopsies implanted under the renal capsule of severely immunodeficient mice formed PDX with 57% efficiency as determined by rising hAAT levels. These findings facilitate a more efficient make‐up of PDX for research into subset‐specific HCC.

## Graphical abstract

The establishment of PDX xenograft models in Hepatocellular carcinoma (HCC) may depend on mouse liver injury. Inducing chronic liver injury in immunodeficient *Fah* ^−/−^ mice through cycling off nitisinone after HCC biopsy implantation, helped produce PDX with 57% efficiency as determined by rising human alpha1‐antitrypsin (hAAT) serum levels.

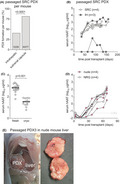

## 1 INTRODUCTION

Hepatocellular carcinoma (HCC) is one of the leading causes of cancer mortality worldwide, largely associated with chronic hepatitis B and C virus (HBV, HCV) infections.^1^ In the U.S. most HCC are caused by HCV infection and alcoholic liver disease (ALD) with a recently rising incidence in HCC secondary to non‐alcoholic fatty liver disease (NAFLD).^2^ The current treatment paradigm consists of curative surgical or radiological interventions for early stage HCC. Tumors that have spread or are not amenable to local interventions are treated with systemic therapies. Sorafenib was the first agent to show a modest benefit for locally advanced and metastatic HCC, while other targeted therapies failed Phase 3 trials at the time of this effort.^3^ More recently, several agents including tyrosine kinase inhibitors^4^, ^5^, ^6^ and immune checkpoint blockade‐based therapies^7^, ^8^, ^9^ have shown improvement in survival outcomes. Genomic diversity among HCC and intra‐tumor heterogeneity challenge clinical trials targeting one or few genomic alterations.^10^ HCC may develop multicentrically from different clones or arise from a single original tumor via intrahepatic metastasis. It is estimated that 22%–79% of synchronous HCC vary clonally, and 12%–66% of single tumors contain intratumor heterogeneity.^11^ As a direct consequence of the tumor heterogeneity, biopsies are necessary for identifying valid biomarkers for HCC and better research models are needed that represent the heterogeneous nature of human HCC.

Human HCC cell lines have been utilized for research, but their translational value has thus far remained limited.^12^ For *in vivo* studies, many mouse models are available that develop murine HCC.^13^ However, existing mouse HCC models poorly recapitulate the human heterogeneity resulting from the aberrant function of multiple molecular pathways.^14^, ^15^, ^16^ Poor reproducibility of murine HCC models has further limited their use.^17^ Therefore, it is imperative to develop experimental models that hold more translational potential, can increase our understanding of human HCC biology and serve as platforms for pre‐clinical testing of novel therapies.

A different strategy to study tumor biology in laboratory animals is to engraft human cancers into immunodeficient mice, commonly referred to as patient‐derived xenografts (PDX).^18^, ^19^ For a number of human cancers, PDX models have been useful to study of cancer biology *in vivo* and predict patient responses to chemotherapeutic regimens.^20^, ^21^, ^22^ To date, PDX have advanced insights into both basic biology and anti‐tumor strategies for breast, colorectal, and several other cancers.^19^ PDX from HCC case series are dominated by HBV‐associated tumors.^23^, ^24^, ^25^, ^26^, ^27^ Establishing PDX from HCC in Western populations has proved more challenging with low success rates.^28^, ^29^, ^30^ With the exception of Zhu et al.^30^ these series compared patient and tumor characteristics rather than animal variables to determine which HCC could establish PDX. We here aimed to quantify to what extent mouse variables influenced PDX formation with HCC biopsy materials obtained from a U.S. patient population. We tested the effects of the murine immunodeficiency, the surgical implantation site, and whether mouse liver injury affected PDX formation.

## 2 MATERIALS AND METHODS

### 2.1 Human subjects

The protocol was reviewed and approved by Memorial Sloan Kettering Cancer Center Institutional Review Board, and by the Rockefeller University Institutional Review Board.

### 2.2 Biopsies

Adult patients of both genders and whose liver lesions were clinically highly suspicious for HCC, required a diagnostic biopsy were included in the study and provided written informed consent for use of tissue samples. Eligible HCC etiologies were HCV infection as determined by HCV serology and/or detectable HCV RT‐PCR; HBV infection as determined by HBV core antibody and/or surface antigen serology, and/or detectable HBV PCR; alcoholic liver disease (ALD) as determined by the history of chronic alcohol use; NAFLD as determined clinically by history, past or current fatty liver on imaging, previous biopsy confirmation and associated metabolic conditions. Biopsies were performed at the interventional radiology department at MSKCC, tumor samples were obtained by image‐guidance technique from primary HCC or metastatic site. Biopsy material was cut into small pieces (~0.5 mm^3^) for implantation.

### 2.3 Animals

Balb/c *Rag2* ^−/−^, NOD *Rag1* ^−/−^ *Il2rg^null^* (NRG) and *Foxn1^nu^* mice were obtained from Jackson Labs (Bar Harbor, Maine). Mice with a targeted disruption in fumaryl acetoacetate hydrolase (*Fah* ^−/−^) were kindly provided by M. Grompe (Oregon Health & Science University) and crossed with *Rag2* ^−/−^ *Il2rg^null^* or with NRG mice.^31^ Immunodeficient *Fah* ^−/−^ were bred and maintained on nitisinone (Yecuris, Tualatin, OR) to prevent liver damage. To induce chronic liver injury immunodeficient *Fah* ^−/−^ mice were cycled off nitisinone after HCC biopsy implantation or kept on continuous nitisinone as non‐liver injury controls. At the time of surgery mice were 6–10 weeks of age with weight varying from 20 g up to 30 g. All procedures were reviewed and approved by Rockefeller University Institutional Review Board and Committee on Use and Care of Animals under protocol number 18063.

### 2.4 Implantation and engraftment monitoring

For intrahepatic implantation, the upper abdomen of mice was shaved and sterilized with iodine. After mice had been anesthetized using isoflurane, a 10–15 mm skin incision was made over the left subcostal margin after which the peritoneum was mobilized. Using a cautery device, 10–15 mm of peritoneum was opened, and the large lobe of the liver was exposed. An ~1 mm opening was created in the center of the lobe and two to three ~0.5 mm^3^ pieces of biopsy material were loaded into the liver. The outflow tract was cauterized to prevent bleeding and then the peritoneum closed with a Vicryl suture and the skin with hemostat wound clips.

For subrenal capsule (SRC) implantation the left flank of mice was shaved and sterilized with iodine. After mice were anesthetized using isoflurane, a 5–7 mm skin incision was made in the left midaxillary plane, after which the peritoneum was mobilized. Using a cautery device 7–8 mm of peritoneum was opened, the left kidney exposed and at the caudal pole. A ~1 mm opening in the capsule was created. Two to three ~0.5 mm^3^ pieces of biopsy material were placed under the kidney capsule and moved cranially. The kidney was mobilized back into the peritoneum, closed with a Vicryl suture and the skin approximated with hemostat wound clips.

Engraftment growth was monitored by quantification of human HCC markers in mouse serum every 3–4 weeks by using commercially available human‐specific ELISA antibodies and protocols as described previously.^31^ If animal health were adversely affected by tumor growth as shown by displaying lethargy, hunched appearance, ruffled fur, and/or cachexia, the experiment was terminated in accordance with the Rockefeller University Institutional Animal Care and Use Committee (IACUC) protocol.

### 2.5 Tissue harvest, passaging, and cryopreservation

Once tumors plateaued as assessed by serum markers or imaging, PDX tissues were harvested for histology and cryopreservation. After CO_2_ asphyxiation the liver and the left kidney were harvested. Tumors were weighed after separation from the liver or kidney. For tumors in which the kidney could not be separated or identified the median left kidney weight for that strain was subtracted from total weight. Tumor tissue was collected in 10% buffered formalin for histology. The remaining tissue was cut into small, 1–2 mm^3^ pieces for immediate passaging or put in hepatocyte freezing medium containing 10% DMSO for long‐term storage at −150ºC.

### 2.6 Histological comparison

Samples were formalin‐fixed, paraffin‐embedded, cut into 5.0 micron sections, and stained with hematoxylin and eosin (H&E). Histopathology was examined under light microscopy.

### 2.7 Statistical analysis

Pearson correlation coefficient was used to assess the linear correlation between tumor weight of mice with serum hAAT among PDX1. A linear mixed model was used to further evaluate the association between tumor weight and serum hAAT from different PDX. At the biopsy level, Fisher's exact test was used to examine the association between immunodeficiency status and implantation site on the success of PDX formation. To account the clustering of repeated binary observations from mice within a PDX, the generalized estimating equations (GEE) logistic regression model was used to examine the association between the factors mentioned above and PDX formation in the mouse level. To look at the association between liver injured and no liver injury on the 18 biopsies implanted under both conditions, the exact McNemar's test was used. Biopsies that were implanted into *Rag2*‐/‐ mice were excluded.

All analyses were carried out in GraphPad Prism Software and SAS 9.4 (SAS Institution, Cary, NC). All *p*‐values were two‐sided and *p*‐value of 0.05 indicated statistical significance.

## 3 RESULTS

### 3.1 Clinical HCC characteristics and overall PDX formation rate

Sixty‐two biopsies were obtained from 60 patients with presumed HCC enrolled in the study. Seventeen samples were excluded from the analysis as they were determined either as cirrhotic liver without HCC (*n* = 4), a non‐HCC cancer (*n* = 5) or because there was no or not enough tissue for implantation (*n* = 8). Of the 45 HCC biopsy specimens that were implanted, 37 were obtained from male patients (82%). Patient demographics and tumor characteristics are summarized in Table 1. Our biopsy material selection highlights the HCC etiological distribution most commonly noted in the United States. The underlying liver disease etiologies of the 45 HCC biopsies were HCV (*n* = 15), alcoholic liver disease (ALD) (*n* = 6), HBV (*n* = 8), NAFLD (*n* = 5), mixed etiology (alcohol and HCV *n* = 2; alcohol and NAFLD *n* = 1; HCV and NAFLD *n* = 1), and no identifiable liver disease etiology or cryptogenic cirrhosis (*n* = 7).

**TABLE 1:** Patients demographics and tumors characteristics of implanted HCC biopsies

Biopsy	Race	Ethnicity	Sex	Site of biopsy	Etiology	Differentiation grade	Growth	AFP, ng/ml
HCC 1	White	Non‐Hispanic	Male	Liver	ALD	Moderate	Yes	4.2
HCC 2	White	Non‐Hispanic	Male	Liver	ALD	Well to moderate	Yes	38.4
HCC 3	Asian	Non‐Hispanic	Female	Liver	HBV	Moderate	Yes	1389
HCC 4	White	Non‐Hispanic	Female	Liver	NAFLD	Poor	Yes	1062
HCC 5	N/A	Non‐Hispanic	Male	Liver	ALD	moderate	Yes	10.1
HCC 6	Asian	Non‐Hispanic	Male	Peritoneum	ALD	Moderate	Yes	27.6
HCC 7	White	Unknown	Male	Liver	NAFLD	N/A	No	263
HCC 8	White	Non‐Hispanic	Male	Perirenal	None	N/A	No	5
HCC 10	Other	Hispanic	Male	Liver	ALD, HCV	Mostly necrotic	Yes	14,121
HCC 13	White	Non‐Hispanic	Male	Liver	HCV	N/A	Yes	949,118
HCC 14	White	Non‐Hispanic	Male	Liver	ALD, NAFLD	Poor	Yes	2719
HCC 15	Asian	Non‐Hispanic	Male	Liver	ALD	Moderate	Yes	208
HCC 16	White	Non‐Hispanic	Male	Liver	HCV	N/A	Yes	1035
HCC 17	White	Non‐Hispanic	Male	Liver	HCV	Moderate	Yes	15.4
HCC 18	White	Non‐Hispanic	Male	Liver	HCV	Well	No	39.6
HCC 19	White	Non‐Hispanic	Male	Liver	HBV, HIV	Moderate	No	6732
HCC 20	White	Non‐Hispanic	Male	Liver	HCV	Moderate	Yes	43
HCC 21	N/A	Non‐Hispanic	Male	Liver	HCV	Well to moderate	Yes	2.9
HCC 22	White	Non‐Hispanic	Female	Liver	None	Moderate	No	42,323
HCC 24	White	Non‐Hispanic	Male	Liver	HCV	Well	No	180
HCC 25	White	Non‐Hispanic	Male	Liver	ALD	N/A	Yes	55,089
HCC 26	White	Non‐Hispanic	Male	Pancreas	HCV	Poor	Yes	1470
HCC 27	White	Non‐Hispanic	Male	Liver	ALD	Moderate	No	381,770
HCC 28	Black	Non‐Hispanic	Male	Liver	HCV	Moderate	Yes	51,687
HCC 29	White	Non‐Hispanic	Female	Paracolic	NAFLD	Poor	No	2293
HCC 31	White	Non‐Hispanic	Female	Abdominal	HBV	N/A	No	16,469
HCC 31 (2)				Peritoneal Node		Poor	Yes	
HCC 32	Black	Non‐Hispanic	Female	Adrenal	None	Well	Yes	355
HCC 32 (2)				Adrenal		N/A	No	
HCC 33	White	Non‐Hispanic	Male	Liver	HBV	N/A	No	6
HCC 35	White	Non‐Hispanic	Male	Liver	HCV	N/A	No	6
HCC 36	Other	Hispanic	Male	Liver	HCV	Moderate	Yes	1,249,574
HCC 40	White	Non‐Hispanic	Female	Liver	Cirrhosis	Moderate	No	228
HCC 42	White	Non‐Hispanic	Male	Liver	Crohns	Poor	No	52
HCC 46	Pacific Islander	Non‐Hispanic	Female	Liver	HCV	Moderate	No	991
HCC 48	Black	Non‐Hispanic	Male	Liver	HBV	N/A	No	143,236
HCC 49	White	Non‐Hispanic	Male	Liver	HCV	Well	No	542
HCC 50	White	Non‐Hispanic	Male	Liver	HCV, HIV	Poor	No	30
HCC 52	White	Non‐Hispanic	Male	Liver	HBV	Moderate	No	4.4
HCC 53	Asian	Non‐Hispanic	Male	Liver	HBV	Moderate	No	125
HCC 56	White	Non‐Hispanic	Male	Liver	None	Moderate	No	2.2
HCC 57	White	Non‐Hispanic	Male	Liver	HCV	Poor	No	74
HCC 59	White	Non‐Hispanic	Male	Liver	HCV	Well to moderate	No	110
HCC 60	White	Non‐Hispanic	Male	Liver	Unknown	Well to moderate	No	1227
HCC 62	White	Non‐Hispanic	Male	Lung	Cirrhosis	N/A	No	263

Abbreviations: ALD, alcoholic liver disease; HBV, hepatitis B virus; HCC, hepatocellular carcinoma; HCV, hepatitis C virus; NAFLD, non‐alcoholic fatty liver disease; N/A, not available.

We found that 20 out of 45 HCC biopsy specimens could establish PDX lines for an overall success rate of 44%. Expectantly, the majority came from male patients (*n* = 16; 80%). These were equally distributed among the four major HCC etiologies in the United States, with 8 (40%) from HCV, 6 (30%) from ALD, 1 (5%) from NAFLD, 2(10%) from HBV, 2 (10%) from alcohol with a secondary liver disease, and 1 (5%) with cryptogenic cirrhosis. These results on a small sample size suggest that HCC biopsy materials from a patient population in the United States can form PDX without a clear preference toward certain liver disease etiologies.

### 3.2 Human alpha1‐antitrypsin is a serum marker of HCC PDX formation in mice

**FIGURE 1:**
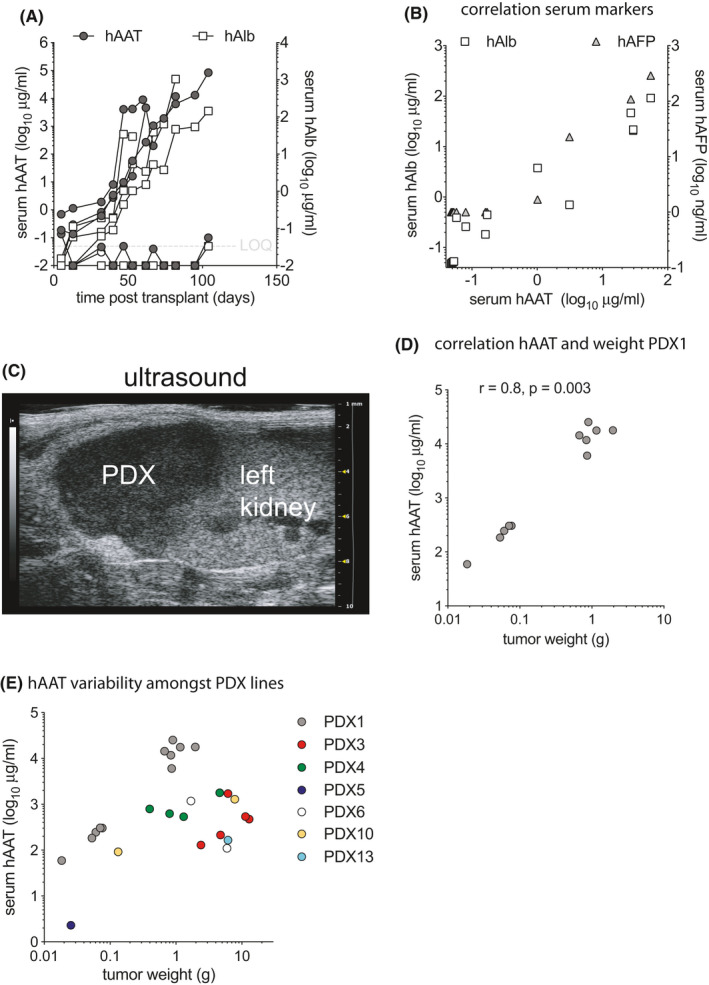
Human AAT is a serum marker for PDX formation. (A) After implantation of HCC1 biopsy materials mice were serially bled and human alpha1‐antitrypsin (hAAT, grey circles) and human albumin (hAlb, white squares) were quantified in mouse serum. Three of five mice showed rising levels of both hAAT and hAlb over time. (B) For PDX3 that secreted hAAT, hAlb, and human alpha‐fetoprotein (hAFP), hAAT levels correlated to the other proteins even though the ratio of hAlb and hAFP varied between mice. (C) A mouse that was transplanted with HCC1 under the SRC and had serum hAAT levels of 1.5 mg/ml was examined by ultrasound. The PDX could readily be visualized as a hypoechoic mass adjacent to the kidney. (D) Tumors from mice that received passaged PDX1 under the SRC were harvested, dissected, and weighed. Serum hAAT levels on the day of harvest correlated with the tumor weight. Pearson correlation coefficient. (E) Tumors from mice transplanted with various PDX lines were weighed and serum hAAT levels quantified. There was no association between tumor weights and hAAT levels across PDX lines

Most HCC tumor specimens have been implanted under the skin of mice, which allows for the detection of large PDX by palpation. When HCC are implanted into other anatomical sites palpation becomes insensitive for small tumors, imaging can be used but is operator‐dependent, labor‐intensive, and costly. We thus set out to test if human markers in mouse serum could identify animals that grew PDX. This would allow for easier PDX monitoring in non‐subcutaneous implantation sites such as intrahepatic (IH) or subrenal capsule (SRC). Serum from mice that were implanted with HCC biopsies were screened for several human markers including albumin (hAlb), α_1_‐antitrypsin (hAAT), transferrin, and α‐fetoprotein (hAFP). As illustrated by PDX1 (Figure 1A), hAAT became detectable in 3 out of 5 mice shortly after HCC implantation and rose over time along with hAlb, while hAFP (not shown) remained undetectable. PDX that released multiple human markers in mouse serum typically did so with similar ratios as illustrated by PDX3 (Figure 1B). Of the first 3 HCC biopsies that were transplanted into mice and that resulted in the rise of any human serum marker, all secreted hAAT. Other markers were inconsistently detectable. From then on serum hAAT was used as a marker for successful PDX formation. Indeed, all mice that contained macroscopic tumors showed rising hAAT levels. And conversely, of mice that were transplanted with HCC biopsy materials and that had unquantifiable (<50 ng/ml) hAAT levels for up to 6 months after surgery, none were found to have a macroscopic PDX. This suggests that serum hAAT, which historically has been investigated as a clinical HCC marker,^32^ is an effective tool to monitor PDX formation.

Next, sonographic detection of PDX was explored in mice with rising hAAT serum levels. In mice with high hAAT levels (e.g., >1 mg/ml for PDX1), PDX in the SRC could be visualized by ultrasound as a hypoechoic mass adjacent to the left kidney (Figure 1C). However, PDX in mice with low serum hAAT levels could not convincingly be visualized by ultrasound either for SRC or IH implanted biopsy materials.

Finally, serum hAAT levels were compared to tumor size. For passaged PDX1 there was a correlation (*r* = 0.8, *p* = 0.003 by Pearson correlation coefficient) between hAAT serum levels and tumor weight (Figure 1D). After accounting for the clustering serum hAAT and tumor weight data from different PDX lines did not show such correlation, which was in line with the highly heterogeneous nature of these tumors (beta = −434.72, *p* = 0.258) (Figure 1E).

These data show that hAAT quantification in mouse serum can serve as an effective tool to screen for PDX formation, and that for an individual PDX line hAAT serum levels correlate with tumor weight.

### 3.3 Only severely immunodeficient mice allow for PDX formation from HCC biopsy material

**FIGURE 2:**
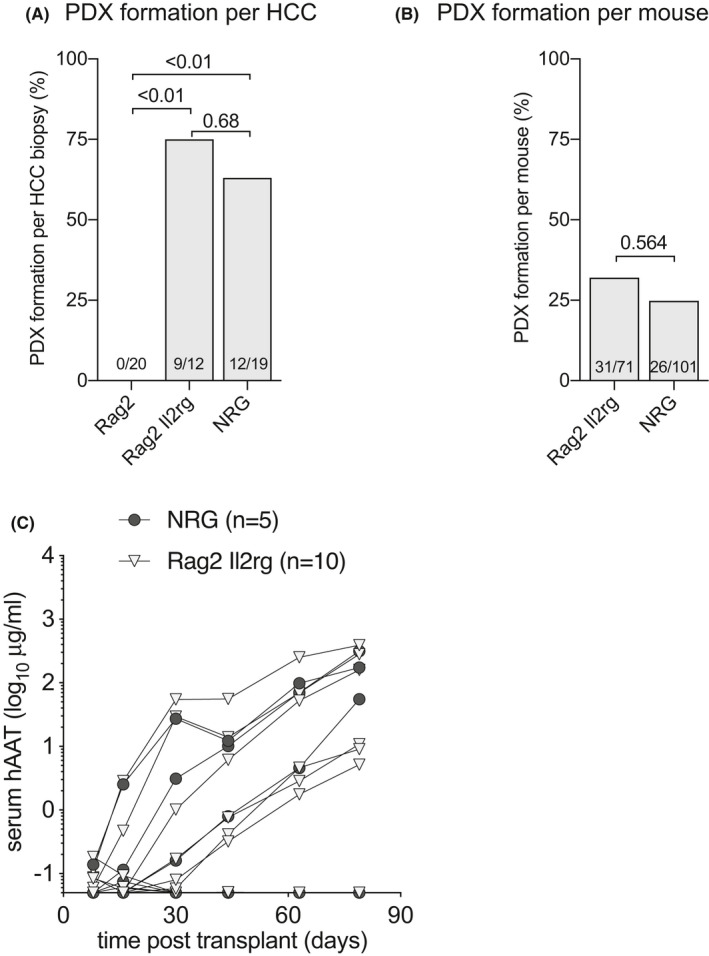
Murine immunodeficiency affects PDX formation. (A) HCC biopsy materials were implanted into groups of mice with three levels of immunodeficiency. PDX formation was counted as positive if at least one mouse per HCC biopsy showed rising hAAT levels. PDX formation rates contrasted sharply between *Rag2* ^−/−^ animals and mice that also lacked the IL‐2Rγ chain, whereas there was no statistically significant difference with NRG mice. Numbers in bars indicate successful PDX formation number over total biopsies implanted, *t*‐test between groups. (B) HCC biopsy materials were implanted in mice with different immunodeficiencies and individual mice with rising hAAT levels were counted positive. *Rag2* ^−/−^ *Il2rg^null^* mice were not statistically better than NRG mice in facilitating PDX formation. T‐test between groups after accounting for clustering. (C) Example of serial hAAT measurements in *Rag2* ^−/−^ *Il2rg^null^* and NRG mice transplanted with HCC3 biopsy material. PDX were established in three out of 5 NRG and 6/10 *Rag2* ^−/−^ *Il2rg^null^* mice based on rising hAAT levels

Innate, humoral and T cell‐mediated rejection all form  major immune barriers to xenotransplantation,^33^ which has led to the widespread use of T and B cell‐deficient mice such as the severe combined immunodeficient (SCID) and recombination activating gene knockout (*Rag* ^−/−^) strains to establish PDX.^34^ We here aimed to test to what extent the immunodeficient background of recipient mice influenced PDX formation. Three immunodeficient strains were used for PDX formation^1^: T and B cell deficient *Rag2* ^−/−^ animals^2^; *Rag2* ^−/−^ mice additionally lacking the interleukin‐2 receptor gamma chain (*Rag2* ^−/−^ *Il2rg^null^*), which results in the absence of natural killer and innate lymphoid cells and some impaired myeloid functions^3^; animals that in addition to *Rag1* ^−/−^ *^^Il2rg^null^* were on the non‐obese diabetic background (NOD *Rag1* ^−/−^ *^^Il2rg^null^* or NRG), which impairs macrophage xenorejection.^35^ Forty‐five biopsy specimens were implanted in these three immunodeficient strains and rising serum hAAT levels were used as a marker for PDX formation. Not a single mouse generated rising hAAT levels with 20 biopsies implanted into *Rag2* ^−/−^ animals. This contrasted with 9 out of 12 (75%) forming a PDX in mice on the *Rag2* ^−/−^ *^^Il2rg^null^* and 7 out of 12 (63%) in NRG mice (Figure 2A). The penetrance of PDX formation, defined as the number of mice that showed rising hAAT levels after HCC biopsy implantation, was higher in animals on the *Rag2* ^−/−^ *Il2rg^null^* (32%) than NRG (25%) immunodeficient background, which was not statistically different (*p* = 0.564) after accounting for the clustering from the different tumors (Figure 2B). Given the limited numbers of PDX there was no association between the immunodeficiency and HCC disease etiology. Mildly immunodeficient *Rag2* ^−/−^ mice prevented PDX formation from all four major HCC etiologies in the U.S. (HCV, ALD, NAFLD, and HBV) whereas *Rag2* ^−/−^ *Il2rg^null^* and NRG mice allowed for PDX establishment from these four etiologies (Table 1). Of the 20 HCC specimens that could form PDX, five biopsy specimens were implanted in both *Rag2* ^−/−^ *Il2rg^null^* and NRG mice. Four grew PDX in both (HCC3 is an example of these four, Figure 2C) whereas one formed PDX only in the NRG immunodeficient background. These data show that the *Rag2* ^−/−^ *Il2rg^null^* immunodeficiency is minimally required to successfully establish PDX from HCC biopsy materials with no benefit of the NOD background.

### 3.4 The HCC biopsy implantation site affects PDX formation rate

**FIGURE 3:**
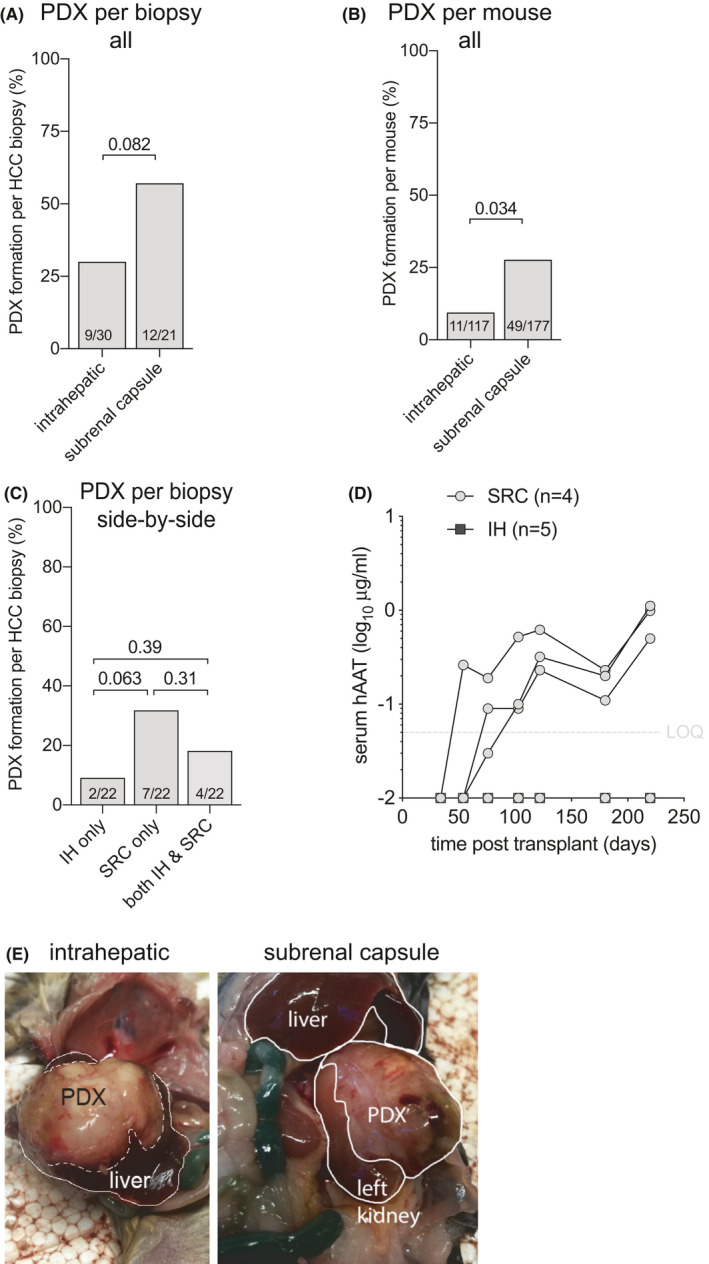
Implantation site affects PDX formation. (A) HCC biopsy materials were implanted subrenal capsule or intrahepatic in groups of mice. PDX formation was counted as positive if at least one mouse per HCC biopsy showed rising hAAT levels. PDX formation rates were numerically higher in the subrenal capsule than intrahepatic location. Numbers in bars indicate successful PDX formation number over total biopsies implanted, *t*‐test between groups. (B) HCC biopsy materials were implanted in subrenal capsule or intrahepatic individual mice with rising hAAT levels were counted as positive. On a per‐mouse basis, PDX formed three‐fold better in the subrenal capsule than intrahepatic. T‐test between groups after accounting for clustering. (C) Twenty‐two HCC biopsies were implanted in groups of mice in the subrenal capsule (SRC) or side‐by‐side in groups of mice intrahepatic (IH), and if at least one mouse per group showed rising hAAT it was counted as positive. The SRC location trended toward higher PDX formation rates than IH location, while only four HCC biopsies established PDX in both anatomical locations. T‐test between groups. (D) Example of serial hAAT measurements in *Rag2* ^−/−^ *Il2rg^null^* and NRG mice transplanted with HCC16 biopsy material. PDX were established in three out of four subrenal capsule (SRC) mice while none of the five intrahepatic (IH) mice showed rising hAAT levels. (E) Example of macroscopic tumors that grew after HCC1 biopsy material was implanted intrahepatic as well as in the subrenal capsule

Most PDX have been established by implanting HCC tissues subcutaneously^23^, ^24^, ^25^, ^26^, ^27^, ^28^, ^29^ while only Zhu et al.^30^ compared intrahepatic (IH) engraftment to subcutaneous implantation. Because of HCC’s arterial blood supply, we hypothesized that the subrenal capsule (SRC) that has historically been used for rapid revascularization of implanted tissue would improve PDX formation rates. To test this hypothesis HCC biopsy materials were implanted SRC and IH, and rising hAAT serum levels were used as a marker for successful PDX formation. Less immunodeficient *Rag2* ^−/−^ mice were excluded from these analyses since this recipient strain precluded all PDX formation. Thirty HCC biopsy specimens were transplanted SRC and 22 biopsy specimens IH into mice on the *Rag2* ^−/−^ *Il2rg^null^* and NRG immunodeficient backgrounds. Overall PDX formation rates appeared higher with SRC (57%) than IH (30%) implantation, which did not reach statistical significance (Figure 3A). Penetrance, defined as how many mice formed PDX after HCC implantation, was indeed higher SRC (28%) than IH (9%) (Figure 3B). Twenty‐two biopsy specimens were transplanted SRC in mice and side‐by‐side IH in other mice. Like the overall numbers, the SRC location trended to be superior for PDX formation (Figure 3C,D) while only four established PDX in both anatomical locations (Figure 3E). For the HCC biopsies that formed PDX in both locations no differences in hAAT kinetics were observed (not shown). Given the small number of PDX lines, there was no clear pattern between HCC disease etiology and location. These data show that HCC biopsy material forms PDX at a 2‐fold higher rate under the renal capsule than in the liver, resulting in an overall 57% success rate in severely immunodeficient mice.

### 3.5 Few HCC require murine liver injury for PDX formation

**FIGURE 4:**
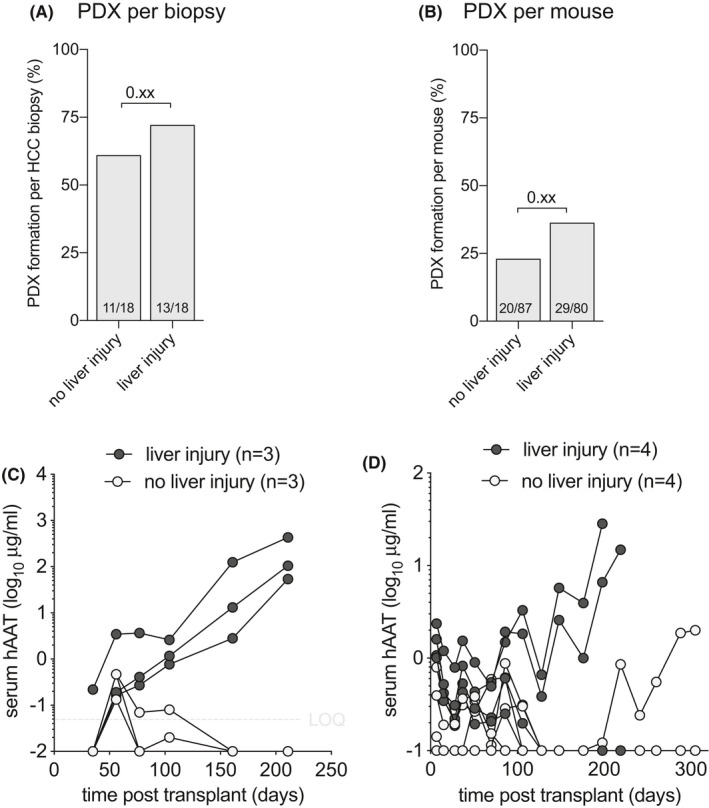
Only two HCC require murine liver injury to establish PDX. (A) HCC biopsy materials were implanted in groups of immunodeficient *Fah* ^−/−^ mice cycled off the drug nitisinone (liver injury) and compared to groups of immunodeficient *Fah* ^−/−^ mice kept on continuous nitisinone or mice without liver injury (no liver injury). PDX formation was counted as positive if at least one mouse per HCC biopsy showed rising hAAT levels. Liver injury resulted in the formation of only 2 (11%) additional PDX lines. Numbers in bars indicate successful PDX formation number over total biopsies implanted, Exact McNemar's test between groups. (B) HCC biopsy materials were implanted in mice with or without liver injury and individual mice with rising hAAT levels were counted as positive. On a per‐mouse basis, PDX formed 1.6‐fold better in mice with liver injury than without liver injury. T‐test between groups after accounting for clustering. (C) Example of serial hAAT measurements in *Fah* ^−/−^ *Rag2* ^−/−^ *Il2rg^null^* mice transplanted with HCC14 biopsy material and cycled off (liver injury) or kept on continuous nitisinone (no liver injury). All three liver injury mice showed rising hAAT levels and none of the mice without liver injury. (D) Example of serial hAAT measurements in *Fah* ^−/−^ NOD*Rag1* ^−/−^ *Il2rg^null^* mice transplanted with HCC2 biopsy material and cycled off (liver injury) or kept on continuous nitisinone (no liver injury). Three months after transplantation two liver injury mice displayed rising hAAT levels followed by one mouse without liver injury four months later

Human hepatocytes can proliferate in response to signals provided by the damaged and/or failing mouse liver, which facilitates the creation of liver chimeric mice. We hypothesized that moderately and well‐differentiated HCC, which are more associated with HCV and ALD than with HBV, would benefit from murine liver injury to form PDX. To test this hypothesis, we implanted HCC biopsy material into immunodeficient *Fah* ^−/−^ mice that underwent liver injury by intermittent withdrawal of the protective drug nitisinone.^36^ These liver injury mice were compared to immunodeficient *Fah* ^−/−^ mice maintained on stable nitisinone or NRG mice without liver injury. We here analyzed the 18 HCC biopsies were implanted both in mice with and without liver injury, excluding *Rag2* ^−/−^ recipients and combining SRC and IH implantation sites. PDX formed in 13/18 (72.2%) in mice with liver injury and 11/18 (61.1%) in animals without liver injury (*p* = 0.21) as defined by rising hAAT serum levels (Figure 4A). The penetrance was also numerically but not statistically significantly higher in mice with liver injury (38%) than mice without liver injury (25%) (Figure 4B). Two biopsies, one from a moderately differentiated HCC caused by HCV and another from a moderately differentiated ALD‐associated HCC formed PDX in mice with liver injury but not in the same immunodeficient *Fah* ^−/−^ mice kept on continuous nitisinone to prevent liver injury (Figure 4C). A third biopsy from a well‐to‐moderately differentiated HCC caused by ALD formed PDX in two out of four mice with liver injury and, only 4 months later, in a syngeneic mouse kept on continuous nitisinone without liver injury (Figure 4D).

These findings show that there is little overall benefit using *Fah* ^−/−^ liver injury mice to establish PDX, yet some moderately and well‐differentiated HCC may better form PDX using this approach.

### 3.6 Passaged PDX are less sensitive to anatomical and immunodeficiency restrictions

**FIGURE 5:**
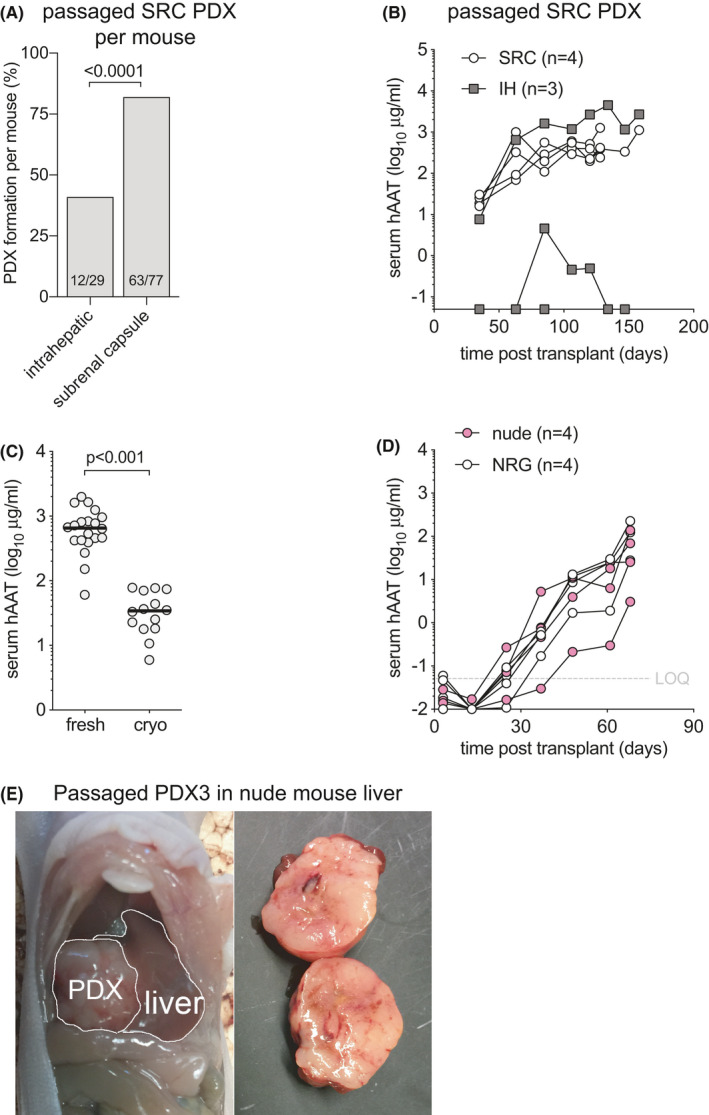
Passaging PDX lessens dependence on location and immunodeficiency. (A) Of the 9 PDX lines that had been established in the subrenal capsule (SRC) and that could be passaged, implantation in the subrenal capsule resulted in a higher number of mice with rising hAAT levels than when these PDX were passaged intrahepatic. T‐test between groups. (B) Example of serial hAAT measurements in NRG mice that were transplanted intrahepatic (IH) or subrenal capsule (SRC) with passaged PDX4 material that had been established SRC. Whereas all four animals that received passaged PDX in the SRC showed rising hAAT levels this tumor could be passaged in only one out of three IH implanted animals. (C) PDX1 that had been established SRC was passaged fresh or after cryopreservation and recovery (cryo) into SRC of NRG mice. On day 36 after transplant, the hAAT levels were 19‐fold higher in animals that received fresh compared to cryopreserved tumor. Symbols individual mice, bars are median, *t*‐test between groups. (D) Example of serial hAAT measurements in *FoxN1^nu^* (nude) and NRG mice transplanted intrahepatic with passaged PDX3 material. All mice showed rising hAAT levels with similar kinetics irrespective of their relative immunodeficiency. (E) Example of a solid tumor that grew after passaged PDX3 was implanted in the liver of an *FoxN1^nu^* (nude) mouse

Once PDX have formed from HCC tissues they generally can readily be passaged into larger cohorts of animals,^23^, ^28^, ^30^ likely because subsets of tumor clones have been selected that are better adapted to the mouse environment.^37^ We serially transplanted 12 PDX lines, 9 of which resulted in rising hAAT serum levels (75%). The penetrance, defined as the number of mice with rising hAAT levels after receiving a passaged PDX, was 85/146 (58%) and contrasted with the lower penetrance of HCC biopsy materials (Figure 2B). We mostly passaged SRC‐derived PDX lines, that showed a modest preference for implantation site. Of the 9 PDX lines that could be passaged, SRC‐derived tumors that were implanted SRC showed rising hAAT levels in 63/77 mice (82%) compared to 12/29 mice (41%) in which these were implanted IH (Figure 5A). This higher penetrance SRC was illustrated by PDX4, in which 4/4 SRC passaged PDX and only 1/3 IH passaged tumors resulted in rising hAAT levels (Figure 5B). Only 3 PDX that grew IH were passaged into new recipients. These PDX could be passaged in 2/6 mice (33%) SRC and in 4/18 mice (22%) IH. These data show that PDX established SRC were more efficiently passaged by SRC than IH implantation, while the reverse may not hold true.

For other cancers, serial PDX passage resulted in the selection of clones that increasingly lose the genetic complexity of the original tumor.^38^ To create a repository of low‐passage PDX that will be useful for future translational studies, non‐passaged PDX would need to be cryopreserved in a way that allows for efficient engraftment after cryo‐recovery. To test how cryopreservation affected engraftment efficiency and kinetics, we transplanted PDX1 that had been cryopreserved for 6 weeks in liquid nitrogen. Cryo‐recovered PDX pieces were implanted in the SRC of 14 NRG mice and compared to 20 NRG mice that had received the same PDX in the SRC without cryopreservation. Thirty‐six days after the respective surgeries hAAT levels in freshly transplanted PDX were 19‐fold higher than in mice that received cryopreserved tissues of comparable weight (Figure 5/span > C). Nevertheless, all cryopreserved tissues showed rising hAAT levels with minimal variation, illustrating that despite cryopreservation this PDX was able to survive the passaging process.

Finally, we tested whether PDX could be passaged into less immunodeficient recipients. Having failed to establish any PDX from HCC biopsy in T and B cell‐deficient *Rag2* ^−/−^ mice, we passaged one PDX into *Rag2* ^−/−^ and four PDX lines into *Foxn1^nu^* (nude) mice that lack only T cells. Interestingly, one out of four *Rag2* ^−/−^ mice showed rising hAAT levels. Two of four PDX lines could be successfully passaged in nude mice. PDX3, which had been passaged through the liver of an NRG mouse, was subsequently able to grow in livers of nude mice with similar hAAT kinetics as in NRG animals (Figure 5D). Upon harvest, solid tumors were observed in the livers of these nude animals (Figure 5E). When these PDX3 from nude livers were further passaged into wild‐type Balb/c mice no rising hAAT levels were observed in any recipients, suggesting that murine T cells retained the ability to reject this tumor.

These combined data show that a majority of PDX can be passaged with a persistent preference for the SRC space. Furthermore, some passaged PDX lines can grow in less immunodeficient mouse strains.

### 3.7 PDX morphologically resemble the HCC biopsy

**FIGURE 6:**
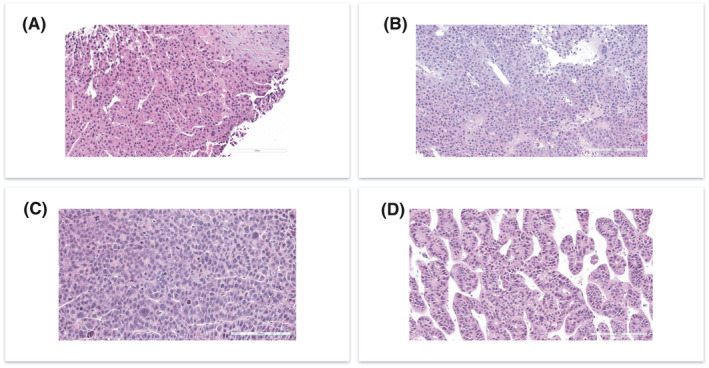
PDX retain the HCC tumor morphology. (A) H&E histology from the patient biopsy. It showed primarily solid growth with vague acinar formation, presence of multinucleated giant cells, mild degree of nuclear pleomorphism, conspicuous mitotic activity, and presence of cytoplasmic granules. Scale bar 200 μm. (B) H&E histology from PDX that was established SRC from patient biopsy shown in (A). PDX maintained the morphology of the original tumor. Scale bar 200 μm. (C) H&E histology from patient biopsy. This tumor showed trabecular growth, prominent sinusoidal rimming and dilated sinusoidal spaces, relatively uniform cytology, and presence of cytoplasmic granules. (D) H&E histology from PDX that was established SRC from patient biopsy shown in (C). PDX maintained similar morphology as seen on patient biopsy

We finally set out to compare PDX histology to clinical histology. Eight PDX lines were recovered and processed for H&E staining. We found that 10 out of 10 (100%) PDX tumors retrieved from 8 HCC primary tumors resembled histopathological characteristics of original human tumor. All tumors were conventional HCC, belonging to the following WHO histologic types: trabecular (2/8 cases), macro‐trabecular (3/8), and solid or compact (3/8). Comparative evaluation of the paired biopsy and PDX samples revealed that the key histological characteristics seen in the biopsies for each individual tumor were recapitulated by the PDX (Figure 6). Specific histological traits seen in paired biopsy and PDX samples are outlined in Table 2. These data show that PDX faithfully recapitulate histological features of the original HCC.

**TABLE 2:** Common histological features between HCC tumors and biopsies from PDX models

PDX	Etiology	Common histological features
PDX1	ALD	Trabecular growth Prominent sinusoidal rimming and dilated sinusoidal spaces Relatively uniform cytology Presence of cytoplasmic granules
PDX3	HBV	Macro‐trabecular growth (focally solid) Moderate degree of nuclear pleomorphism Presence of cytoplasmic inclusions and globules
PDX4	NAFLD	Primarily solid grow with vague acinar formation Presence of multinucleated giant cells (red arrows) Mild degree of nuclear pleomorphism Conspicuous mitotic activity Presence of cytoplasmic granules
PDX6	NAFLD	Macrotrabecular growth Conspicuous sinusoidal rimming with dilated sinusoidal spaces Mild degree of nuclear pleomorphism Conspicuous mitotic activity Presence of cytoplasmic inclusions and vacuoles
PDX10	HCV	Solid growth (focally macro‐trabecular) Moderate degree of nuclear pleomorphism Focal presence of multinucleated tumor cells Focal presence of cytoplasmic inclusions
PDX13	HCV	Solid growth (focally macrotrabecular) Mild degree of nuclear pleomorphism Presence of cytoplasmic inclusions and vacuoles
PDX13	HCV	Solid and focal macrotrabecular growth High‐grade cytology with conspicuous mitotic activity
PDX21	HCV	Trabecular growth with small acinar formation Relatively uniform cytology with smaller than usual nuclei and small/ inconspicuous nucleoli

## 4 DISCUSSION

We identified a human serum marker, hAAT, to screen for PDX formation. It was detectable in all mice that contained tumors upon dissection and no tumors were found in mice that had undetectable hAAT in their serum. We then used rising hAAT as a surrogate marker for PDX formation. This approach comes with some advantages and caveats. hAAT could be detected early after HCC biopsy implantation in many animals (e.g. Figure 4D) even when it disappeared over time, suggesting that some HCC biopsy material may have survived after implantation but did not grow. In our presented herein experiments, rising hAAT was more sensitive to determine tumor growth than ultrasonography, which was inconsistent between measurements, operator‐dependent, time‐consuming and costly. Several animals with rising hAAT that underwent early ultrasonography and in which no tumor could convincingly be visualized eventually grew macroscopically identifiable PDX. The major caveat of using hAAT as a marker for PDX growth is that it can be expressed by other cell types than HCC. In healthy individuals, hepatocytes are the main source of AAT but it can be secreted by other cell types of myeloid lineage. It is conceivable that non‐HCC human tissues produced hAAT in our mice, as has been shown for certain non‐Hodgkin lymphoma subclasses.^39^ Importantly, the PDX that were harvested in our series all matched patient HCC histology and we did not observe B cell lymphomas as reported by others.^29^ However, it is conceivable that some of our mice with rising hAAT contained undetected lymphomas or other non‐HCC xenografts.

The molecular analysis of large numbers of HCC has allowed definitions of distinct classes. One proposed classification divides HCC into two broad phenotypes. The first is associated with cell cycle progression and proliferation and termed the “proliferative” class, which is associated with HBV infection. A second phenotype has retained many features resembling normal hepatocyte physiology, termed “non‐proliferative” class, which are enriched for HCV and ALD patients.^40^, ^41^ These two classes correspond to previously well‐recognized clinical phenotypes. High AFP, EpCAM positive, poorly differentiated tumors predominantly cluster in the proliferative subclass. The low AFP, well‐to‐moderately differentiated tumors are enriched in the non‐proliferative class.^42^ Until recently most PDX series were created with HBV‐associated HCC materials, suggesting that non‐proliferative class HCC poorly established PDX. Our results confirm recent studies^28^, ^30^ that the liver disease etiology in Western populations does not strongly predict whether HCC can form PDX. We found that the major histological subtypes could all establish PDX, and patient biopsies corresponded with PDX histology. This is in line with observations by others and further supported by transcriptomic profiling where gene expression of PDX was closer to the original HCC rather than between different PDX lines.^23^, ^24^, ^28^, ^30^ These and our data illustrate that Western population HCC of all four etiologies can establish PDX that retain the original tumor histology and transcriptome profile.

Based on rising hAAT levels we were able to establish PDX at higher rates than have thus far been reported, particularly for non‐proliferative HCC in Western patient populations.^28^, ^30^ This may be due to a combination of factors. We combined a high‐volume clinical practice at Memorial Sloan Kettering Cancer Center with a laboratory at Rockefeller University that has long‐standing experience with human hepatocyte xenotransplantation into mice. The proximity of both centers allowed for transport and transplantation of HCC material into mice within 1–2 hours following the clinical biopsy procedure. Our prior experience with human hepatocytes taught us that prolonged storage on ice impaired engraftment and that cryopreservation resulted in ~90% lower humanization. Although HCC tissue may be more robust than primary human hepatocyte suspensions, the short extracorporeal time may have benefited PDX formation rates. Another difference that clearly contributed to a higher success rate was the use of the subrenal capsule (SRC) as an implantation site. Other HCC PDX series exclusively used subcutaneous implantation and only Zhu et al.^30^ compared subcutaneous to intrahepatic implantation. For other cancers, PDX formation rates varied across different anatomical sites. For example, implantation under the renal capsule yielded a 90% engraftment rate for non‐small cell lung cancers as opposed to 25% engraftment subcutaneously.^43^ Our original hypothesis that orthotopic, that is, mostly intrahepatic, implantation would improve PDX formation rates proved false. Although some HCC could form intrahepatic PDX, SRC implantation was twice as successful. Because of its arterial blood supply in patients, we speculate that some HCC require a highly perfused SRC microenvironment to establish PDX. This cannot consistently be achieved by randomly inserting biopsy material into the mouse liver. Our result contrasts with breast cancer implantation in the mouse mammary fat pad resulting in 37% PDX formation, which was higher than had been historically observed after subcutaneous implantation.^20^

Non‐proliferative subclass HCC share gene expression pathways with hepatocytes. Normal hepatocytes in a healthy liver have a low mitotic rate but during liver injury go through repeated cell divisions. The factors that drive hepatocyte proliferation as part of physiological liver regeneration are complex and involve many growth factors, inflammatory cytokines, and other pathways.^44^ The majority of HCC develop in patients with cirrhosis, which typically occurs after decades of injury and compensatory hepatocyte proliferation. The mechanisms for the association between cirrhosis and HCC development are poorly understood and may involve the release of growth factors and cytokines that are normally involved in liver regeneration, e.g., fibroblast growth factors and hepatocyte growth factor (HGF). We hypothesized that even though they have acquired mutations for malignant transformation during years of hepatocyte proliferation, non‐proliferative subclass HCC would remain dependent on growth factors from a failing liver to establish PDX. The clinical observations that more advanced liver failure correlates with more aggressive HCC growth^45^ and that patients with HCC have higher serum HGF levels than cirrhotic control patients^46^ supported this hypothesis. Yet our results suggest that murine liver injury is not required for the majority of HCC to establish PDX. As such these tumors have lost their dependence on growth factors to continue proliferating. These results confirm recent data by Zhu et al. who found no statistically significant enhancement of HCC PDX formation in immunodeficient *Fah* ^−/−^ liver injury animals.^30^ Interestingly they observed faster PDX growth in mice that had undergone partial hepatectomy, which suggests that in their first weeks after HCC implantation growth signals from failing mouse liver can benefit PDX establishment. Given the additional costs and technical challenges working with liver injury models, it will be important to better characterize which HCC subclasses require such an approach to form PDX.

Xenografts can be rejected through hyper‐acute, acute and chronic mechanisms orchestrated by humoral, NK cell and T cell‐mediated immunity. It is for these reasons that most PDX studies use broadly severely immunodeficient mice, typically NRG or similar NOD SCID *Il2rg^null^* animals.^34^, ^47^ For HCC PDX there has been no comparison between immunodeficient mouse strains, and our observation that *Rag2* ^−/−^ animals were able to prevent PDX formation cautions against the use of mice with intact NK and other innate immune cell populations. Serial passaging of PDX can select for clones that are more resistant to murine rejection as illustrated by our PDX3 passaging into nude mice. Whether such an approach is more meaningful for immuno‐oncology research than combining PDX models with mice reconstituted with human cells will require further investigations.^48^, ^49^ Both approaches come with important immunological limitations, in addition to the technical challenges associated with harvesting hematopoietic stem cells from patients with advanced HCC and the short lifespan of mice that are transplanted with patient‐derived lymphocytes. For these reasons, substantial effort and innovation will be required to advance PDX studies for routine immuno‐oncology research.

In conclusion, our work is a major advance based on the high (57%) PDX formation rate when fresh HCC biopsy material is implanted in the SRC of severely immunodeficient mice. This protocol can readily be replicated with commercially available mouse strains and thus creates opportunities to advance human HCC research in several directions. It will facilitate the creation of large numbers of PDX lines from non‐proliferative HCC for studies into the numerous pathways that have been implicated in tumor growth. It will advance long‐term goals to combine PDX with autologous human immune system mice, or with costly human liver chimeras infected with viral hepatitis or with fatty liver disease. And in the longer term, it will help advance the promise of creating personalized tumor models to test therapies for patients.

## ETHICS STATEMENT

The protocol was reviewed and approved by Memorial Sloan Kettering Cancer Center Institutional Review Board and by the Rockefeller University Institutional Review Board.

## CONFLICT OF INTEREST

The authors declare no conflict of interest.

## Data Availability

Data is available upon request with a justified explanation.

## References

[1] El‐Serag HB . Hepatocellular carcinoma. N Engl J Med. 2011;365:1118‐1127. 21992124 10.1056/NEJMra1001683

[2] Charlton MR , Burns JM , Pedersen RA , Watt KD , Heimbach JK , Dierkhising RA . Frequency and outcomes of liver transplantation for nonalcoholic steatohepatitis in the United States. Gastroenterology. 2011;141:1249‐1253. 21726509 10.1053/j.gastro.2011.06.061

[3] Llovet JM , Ricci S , Mazzaferro V , et al. Sorafenib in advanced hepatocellular carcinoma. N Engl J Med. 2008;359:378‐390. 18650514 10.1056/NEJMoa0708857

[4] Kudo M , Finn RS , Qin S , et al. Lenvatinib versus sorafenib in first‐line treatment of patients with unresectable hepatocellular carcinoma: a randomised phase 3 non‐inferiority trial. Lancet. 2018;391:1163‐1173. 29433850 10.1016/S0140-6736(18)30207-1

[5] Bruix J , Qin S , Merle P , et al. Regorafenib for patients with hepatocellular carcinoma who progressed on sorafenib treatment (RESORCE): a randomised, double‐blind, placebo‐controlled, phase 3 trial. Lancet. 2017;389:56‐66. 27932229 10.1016/S0140-6736(16)32453-9

[6] Abou‐Alfa GK , Meyer T , Cheng AL , et al. Cabozantinib in patients with advanced and progressing hepatocellular carcinoma. N Engl J Med. 2018;379:54‐63. 29972759 10.1056/NEJMoa1717002 PMC7523244

[7] El‐Khoueiry AB , Sangro B , Yau T , et al. Nivolumab in patients with advanced hepatocellular carcinoma (CheckMate 040): an open‐label, non‐comparative, phase 1/2 dose escalation and expansion trial. Lancet. 2017;389:2492‐2502. 28434648 10.1016/S0140-6736(17)31046-2 PMC7539326

[8] Zhu AX , Finn RS , Edeline J , et al. Pembrolizumab in patients with advanced hepatocellular carcinoma previously treated with sorafenib (KEYNOTE‐224): a non‐randomised, open‐label phase 2 trial. Lancet Oncol. 2018;19:940‐952. 29875066 10.1016/S1470-2045(18)30351-6

[9] Finn RS , Qin S , Ikeda M , et al. Atezolizumab plus bevacizumab in unresectable hepatocellular carcinoma. N Engl J Med. 2020;382:1894‐1905. 32402160 10.1056/NEJMoa1915745

[10] Caruso S , O'Brien DR , Cleary SP , Roberts LR , Zucman‐Rossi J . Genetics of HCC: novel approaches to explore molecular diversity. Hepatology. 2021;73:14‐26. 32463918 10.1002/hep.31394

[11] Lu LC , Hsu CH , Hsu C , Cheng AL . Tumor heterogeneity in hepatocellular carcinoma: facing the challenges. Liver Cancer. 2016;5:128‐138. 27386431 10.1159/000367754 PMC4906428

[12] Molina‐Sanchez P , Lujambio A . Experimental models for preclinical research in hepatocellular carcinoma. In: Hoshida Y , ed. Hepatocellular Carcinoma: Translational Precision Medicine Approaches. Cham (CH); 2019:333‐358. 32078271

[13] Bakiri L , Wagner EF . Mouse models for liver cancer. Mol Oncol. 2013;7:206‐223. 23428636 10.1016/j.molonc.2013.01.005 PMC5528415

[14] Cho K , Ro SW , Seo SH , et al. Genetically engineered mouse models for liver cancer. Cancers. 2020;12:14. 10.3390/cancers12010014 PMC7016809 31861541

[15] Dow M , Pyke RM , Tsui BY , et al. Integrative genomic analysis of mouse and human hepatocellular carcinoma. Proc Natl Acad Sci U S A. 2018;115:E9879‐E9888. 30287485 10.1073/pnas.1811029115 PMC6196518

[16] Yim SY , Lee JS . Genomic perspective on mouse liver cancer models. Cancers. 2019;11:1648. 10.3390/cancers11111648 PMC6895968 31731480

[17] Shlomai A , de Jong YP , Rice CM . Virus associated malignancies: the role of viral hepatitis in hepatocellular carcinoma. Semin Cancer Biol. 2014;26:78‐88. 24457013 10.1016/j.semcancer.2014.01.004 PMC4048791

[18] Siolas D , Hannon GJ . Patient derived tumor xenografts: transforming clinical samples into mouse models. Can Res. 2013;73:5315‐5319. 10.1158/0008-5472.CAN-13-1069 PMC3766500 23733750

[19] Tentler JJ , Tan AC , Weekes CD , et al. Patient‐derived tumour xenografts as models for oncology drug development. Nat Rev Clin Oncol. 2012;9:338‐350. 22508028 10.1038/nrclinonc.2012.61 PMC3928688

[20] DeRose YS , Wang G , Lin Y‐C , et al. Tumor grafts derived from women with breast cancer authentically reflect tumor pathology, growth, metastasis and disease outcomes. Nat Med. 2011;17:1514‐1520. 22019887 10.1038/nm.2454 PMC3553601

[21] Das Thakur M , Pryer NK , Singh M . Mouse tumour models to guide drug development and identify resistance mechanisms. J Pathol. 2014;232:103‐111. 24122209 10.1002/path.4285

[22] Hidalgo M , Amant F , Biankin AV , et al. Patient derived xenograft models: an emerging platform for translational cancer research. Cancer Discov. 2014;4:998‐1013. 25185190 10.1158/2159-8290.CD-14-0001 PMC4167608

[23] Gu Q , Zhang B , Sun H , et al. Genomic characterization of a large panel of patient‐derived hepatocellular carcinoma xenograft tumor models for preclinical development. Oncotarget. 2015;6:20160‐20176. 26062443 10.18632/oncotarget.3969 PMC4652995

[24] Hu B , Li H , Guo W , et al. Establishment of a hepatocellular carcinoma patient‐derived xenograft platform and its application in biomarker identification. Int J Cancer. 2020;146:1606‐1617. 31310010 10.1002/ijc.32564

[25] Huynh H , Soo KC , Chow PK , Panasci L , Tran E . Xenografts of human hepatocellular carcinoma: a useful model for testing drugs. Clin Cancer Res. 2006;12:4306‐4314. 16857806 10.1158/1078-0432.CCR-05-2568

[26] Liu J , Chen S , Zou Z , Tan D , Liu X , Wang X . Pathological pattern of intrahepatic HBV in HCC is phenocopied by PDX‐derived mice: a novel model for antiviral treatment. Transl Oncol. 2019;12:1138‐1146. 31202090 10.1016/j.tranon.2019.05.006 PMC6581976

[27] Xin H , Wang K , Hu G , et al. Establishment and characterization of 7 novel hepatocellular carcinoma cell lines from patient‐derived tumor xenografts. PLoS One. 2014;9:e85308. 24416385 10.1371/journal.pone.0085308 PMC3887059

[28] Blumer T , Fofana I , Matter MS , et al. Hepatocellular carcinoma xenografts established from needle biopsies preserve the characteristics of the originating tumors. Hepatol Commun. 2019;3:971‐986. 31334445 10.1002/hep4.1365 PMC6601318

[29] Chen K , Ahmed S , Adeyi O , Dick JE , Ghanekar A . Human solid tumor xenografts in immunodeficient mice are vulnerable to lymphomagenesis associated with Epstein‐Barr virus. PLoS One. 2012;7:e39294. 22723990 10.1371/journal.pone.0039294 PMC3377749

[30] Zhu M , Li L , Lu T , et al. Uncovering biological factors that regulate hepatocellular carcinoma growth using patient‐derived xenograft assays. Hepatology. 2020;72:1085‐1101. 31899548 10.1002/hep.31096 PMC7332388

[31] de Jong YP , Dorner M , Mommersteeg MC , et al. Broadly neutralizing antibodies abrogate established hepatitis C virus infection. Sci Transl Med. 2014;6:254ra129. 10.1126/scitranslmed.3009512 PMC4312107 25232181

[32] Lee HB , Yoo OJ , Ham JS , Lee MH . Serum alpha 1‐antitrypsin in patients with hepatocellular carcinoma. Clin Chim Acta. 1992;206:225‐230. 1376649 10.1016/0009-8981(92)90092-5

[33] Lu T , Yang B , Wang R , Qin C . Xenotransplantation: current status in preclinical research. Front Immunol. 2019;10:3060. 32038617 10.3389/fimmu.2019.03060 PMC6989439

[34] Okada S , Vaeteewoottacharn K , Kariya R . Application of highly immunocompromised mice for the establishment of patient‐derived xenograft (PDX) models. Cells. 2019;8:889. 10.3390/cells8080889 PMC6721637 31412684

[35] Takenaka K , Prasolava TK , Wang JC , et al. Polymorphism in Sirpa modulates engraftment of human hematopoietic stem cells. Nat Immunol. 2007;8:1313‐1323. 17982459 10.1038/ni1527

[36] Grompe M , Lindstedt S , Al‐Dhalimy M , et al. Pharmacological correction of neonatal lethal hepatic dysfunction in a murine model of hereditary tyrosinaemia type I. Nat Genet. 1995;10:453‐460. 7545495 10.1038/ng0895-453

[37] Ben‐David U , Ha G , Tseng YY , et al. Patient‐derived xenografts undergo mouse‐specific tumor evolution. Nat Genet. 2017;49:1567‐1575. 28991255 10.1038/ng.3967 PMC5659952

[38] Nguyen LV , Cox CL , Eirew P , et al. DNA barcoding reveals diverse growth kinetics of human breast tumour subclones in serially passaged xenografts. Nat Commun. 2014;5:5871. 25532760 10.1038/ncomms6871 PMC4284657

[39] Duplantier MM , Lamant L , Sabourdy F , de Reynies A , Delsol G , Espinos E . Serpin A1 is overexpressed in ALK+ anaplastic large cell lymphoma and its expression correlates with extranodal dissemination. Leukemia. 2006;20:1848‐1854. 16900211 10.1038/sj.leu.2404352

[40] Hoshida Y , Nijman SMB , Kobayashi M , et al. Integrative transcriptome analysis reveals common molecular subclasses of human hepatocellular carcinoma. Can Res. 2009;69:7385‐7392. 10.1158/0008-5472.CAN-09-1089 PMC3549578 19723656

[41] Calderaro J , Ziol M , Paradis V , Zucman‐Rossi J . Molecular and histological correlations in liver cancer. J Hepatol. 2019;71:616‐630. 31195064 10.1016/j.jhep.2019.06.001

[42] Yamashita T , Forgues M , Wang W , et al. EpCAM and α‐Fetoprotein expression defines novel prognostic subtypes of hepatocellular carcinoma. Can Res. 2008;68:1451‐1461. 10.1158/0008-5472.CAN-07-6013 18316609

[43] Dong X , Guan J , English JC , et al. Patient‐derived first generation xenografts of non‐small cell lung cancers: promising tools for predicting drug responses for personalized chemotherapy. Clin Cancer Res. 2010;16:1442‐1451. 20179238 10.1158/1078-0432.CCR-09-2878

[44] Stanger BZ . Cellular homeostasis and repair in the mammalian liver. Annu Rev Physiol. 2015;77:179‐200. 25668020 10.1146/annurev-physiol-021113-170255 PMC5830102

[45] Huo TI , Wu JC , Lin HC , et al. Determination of the optimal model for end‐stage liver disease score in patients with small hepatocellular carcinoma undergoing loco‐regional therapy. Liver Transpl. 2004;10:1507‐1513. 15558587 10.1002/lt.20310

[46] Shiota G , Okano J , Kawasaki H , Kawamoto T , Nakamura T . Serum hepatocyte growth factor levels in liver diseases: clinical implications. Hepatology. 1995;21:106‐112. 7806142

[47] Walsh NC , Kenney LL , Jangalwe S , et al. Humanized mouse models of clinical disease. Annu Rev Pathol. 2017;12:187‐215. 27959627 10.1146/annurev-pathol-052016-100332 PMC5280554

[48] Yao LC , Aryee KE , Cheng M , Kaur P , Keck JG , Brehm MA . Creation of PDX‐bearing humanized mice to study immuno‐oncology. Methods Mol Biol. 2019;1953:241‐252. 30912026 10.1007/978-1-4939-9145-7_15

[49] Zhao Y , Shuen TWH , Toh TB , et al. Development of a new patient‐derived xenograft humanised mouse model to study human‐specific tumour microenvironment and immunotherapy. Gut. 2018;67:1845‐1854. 29602780 10.1136/gutjnl-2017-315201 PMC6145285

